# Cross‐kingdom RNA interference as a unifying mechanism in plant–microbe interactions

**DOI:** 10.1111/nph.71409

**Published:** 2026-07-09

**Authors:** Summia Gul, An‐Po Cheng

**Affiliations:** ^1^ Unit of Plant Molecular Cell Biology, Institute for Biology I RWTH Aachen University Worringerweg 1 Aachen 52056 Germany; ^2^ Max Planck Institute of Molecular Plant Physiology Am Muehlenberg 1 Potsdam‐Golm D‐14476 Germany

**Keywords:** cross‐kingdom RNAi, induced systemic resistance, *PRIM2*, ROS‐mediated immunity, small RNA

## Abstract

This article is a Commentary on Hernández‐Hernández *et al*. (2026), **251**: 3489–3506.

## From virulence to mutualism: ckRNAi is a conserved communication strategy

Small RNAs (sRNAs) mediate gene silencing through RNA interference (RNAi). These sRNAs include small interfering RNAs (siRNAs) and microRNAs (miRNAs) that regulate gene expression and immunity in plant–microbe interactions (Huang *et al*., 2019). Cross‐kingdom RNA interference (ckRNAi) refers to RNAi between organisms belonging to different kingdoms of life. A well‐studied example of ckRNAi is the bidirectional exchange of sRNAs between plants and their pathogens where sRNAs silence host defence genes or pathogen virulence factors. Thus, ckRNAi is part of the molecular dialogue between plants and associated microbes. Pioneering studies indicated that fungal pathogens such as *Botrytis cinerea* deliver sRNAs to hijack the host silencing machinery and to suppress immunity (Weiberg *et al*., 2013), while plants, in turn, export sRNAs to silence the virulence genes of pathogens (Wang *et al*., 2016). ckRNAi has lately been found in symbiotic interactions, that is in arbuscular mycorrhizas and ectomycorrhizas. In arbuscular mycorrhizas, fungal sRNAs from *Rhizophagus irregularis* target host transcription factors that restrict symbiosis, thereby facilitating fungal accommodation (Silvestri *et al*., 2025). Studies have also demonstrated that bidirectional RNA exchange enhances symbiotic efficiency by modulating gene expression in both partners (Usländer *et al*., 2026). The ectomycorrhizal fungus *Pisolithus microcarpus* deploys a symbiosis‐induced miRNA in colonisation of *Eucalyptus grandis* (Wong‐Bajracharya *et al*., 2022). Therefore, sRNAs regulate host transcriptional networks in both mutualistic and pathogenic plant–fungal relations.

*RNA‐based communication is a widespread and versatile process that enables organisms to dynamically regulate their interactions*.

## The discovery of *Ta_sRNA1* and *PRIM2*

The study of Hernández‐Hernández *et al*. (2026; pp. 3489–3506), published in this issue of *New Phytologist*, represents a further expansion of this paradigm. An essential finding of this work is that the characterisation of sRNA‐based communication within the tripartite interaction between *Arabidopsis thaliana*, the fungus *Trichoderma atroviride*, and the fungal pathogen *B. cinerea*. *Trichoderma atroviride* has plant‐beneficial effects by protecting plants from pathogens. The molecular mechanisms underlying this protective effect have, however, remained largely elusive.

**Fig. 1:**
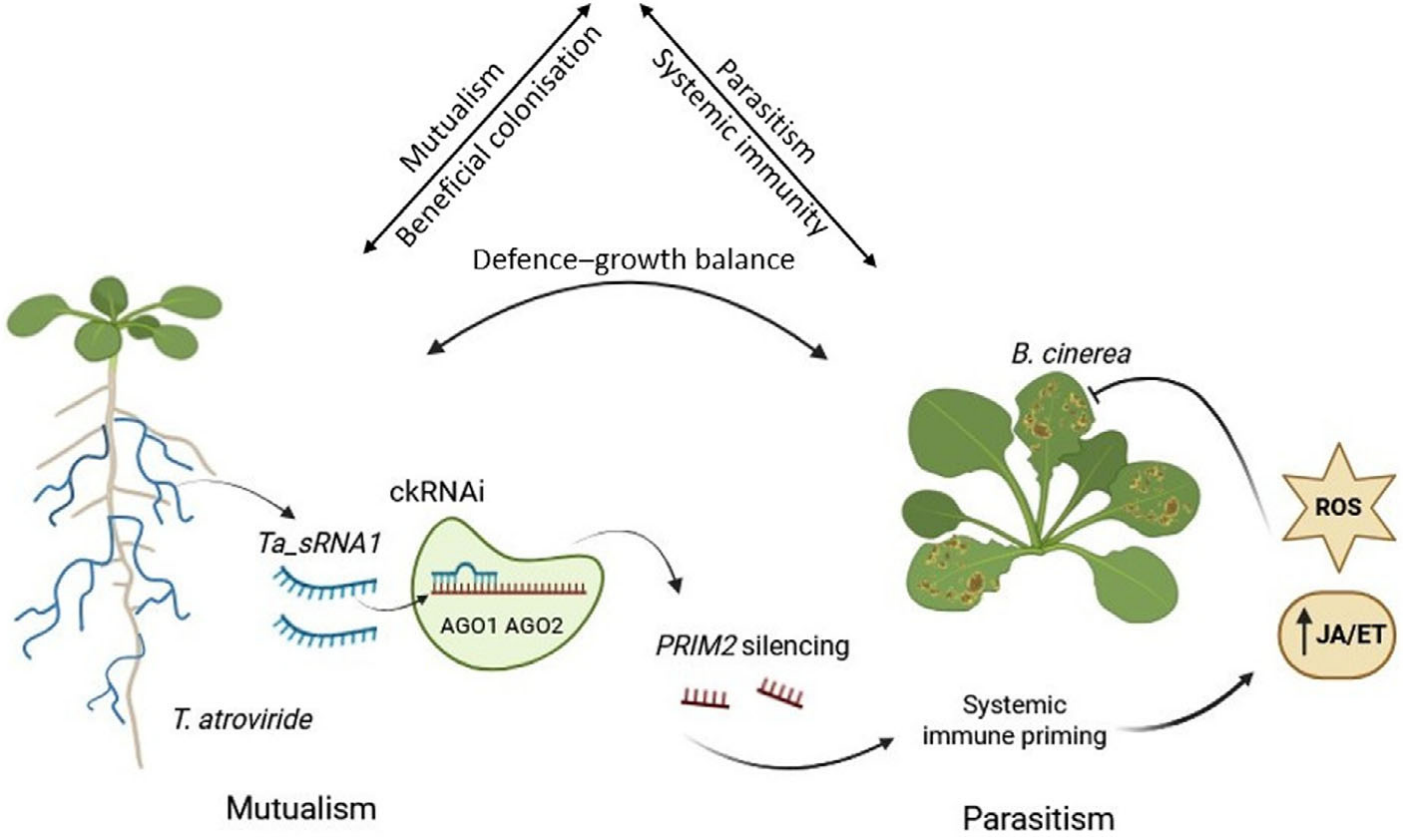
Cross‐kingdom RNA interference (ckRNAi)‐mediated interactions in a tripartite symbiont–plant–pathogen system. The small RNA *Ta_sRNA1*, transferred from the beneficial fungus *Trichoderma atroviride*, silences its target gene *PRIM2* in *Arabidopsis thaliana* roots via association with Argonaute (AGO) protein complexes in a process called ckRNAi. Silencing of the susceptibility gene *PRIM2* induces systemic immune priming, characterised by the activation of jasmonic acid/ethylene (JA/ET) pathway marker genes and the accumulation of reactive oxygen species (ROS). This systemic response leads to localised cell death and enhanced resistance against *Botrytis cinerea* (as observed under natural conditions). Thus, beneficial colonisation mediated by a symbiont‐derived sRNA promotes systemic immunity while contributing to the balance between plant growth and defence.

To study this, the authors sequenced sRNA of *A. thaliana* roots colonised by *T. atroviride*, identifying a set of fungal sRNAs, among which *Ta_sRNA1* was the most abundant and dynamically regulated. *Ta_sRNA1* is a sRNA effector that enters *A. thaliana* root cells and hijacks host AGO1/2 complexes to silence the *PRIM2* encoding DNA primase that acts as a susceptibility factor for colonisation by *T. atroviride* (Fig. 1). Transient co‐expression assays in *Nicotiana benthamiana*, together with mutational analysis of the sRNA binding site, demonstrated repression of *PRIM2* at both transcript and protein levels. The fact that *T. atroviridae* silences a host susceptibility gene (S‐gene) for its own colonisation seems, at first glance, counterintuitive. *Ta_sRNA1* shows a biphasic accumulation pattern, indicating temporally distinct roles during the initial establishment and subsequent stabilisation of the interaction.

The identification of *PRIM2* as a S‐gene is particularly intriguing. The authors demonstrate by mutant and overexpression experiments that suppression of *PRIM2* enables colonisation, while placing limits on over‐colonisation by *T. atroviride* and enhancing resistance to the pathogenic fungus *B. cinerea*. These multiple roles underscore a balance between defence and compatibility. How exactly a DNA primase affects microbial accommodation remains obscure and the role of S‐genes in symbiosis is unclear. Targeting of *PRIM2* indicates that beneficial microbes use the same host vulnerabilities as pathogens, but in a controllable manner that benefits both parties. This suppression allows colonisation without triggering excessive immune responses. The broader implication is that ckRNAi‐mediated silencing of S‐genes may represent a common strategy across diverse plant–microbe interactions, fundamentally advancing our understanding of symbiont‐driven plant protection.

## Argonaute proteins serve as central hubs in cross‐kingdom communication

For foreign sRNAs to affect the host, they require compatible machinery, and Argonaute (AGO) proteins serve as hubs of this communication. AGO proteins are mediators of RNAi; they bind sRNAs and use them as guides to recognise and silence complementary target genes. Hernández‐Hernández *et al*. discovered by AGO immunoprecipitation that *Ta_sRNA1* associates with both AGO1 and AGO2 in *A. thaliana*, where AGO1 is the primary mediator of *PRIM2* repression. The biochemical evidence is supported by genetic data: *PRIM2* expression is differentially affected in *ago1* and *ago2* mutants, indicating that both AGOs contribute to its regulation. AGO1 is typically associated with miRNA‐like gene silencing, while AGO2 is often linked to stress responses (Brosseau *et al*., 2020; Zhu *et al*., 2022). *Ta_sRNA1*'s preferential association with AGO1 suggests that beneficial microbes can take advantage of classical regulatory pathways.

ckRNAi communication is typically bidirectional. Recent work by Cheng *et al*. (2025) revealed the role of fungal AGOs, indicating these proteins contribute actively to the exchange and function of sRNAs between kingdoms. Thus, both partners possess the machinery to send, receive, and process RNA signals. The emerging picture is that ckRNAi is highly coordinated and sRNAs are selectively packaged into AGO complexes and distributed across species.

## Systemic priming via modulation of host hormonal networks and redox homeostasis

*Ta_sRNA1* regulates *T. atroviride*‐mediated induced systemic resistance (ISR) in plants by modulating hormone‐based defence signalling. Ectopic expression of *Ta_sRNA1* disrupts the balance between jasmonic acid (JA)/ethylene‐ and salicylic acid (SA)‐dependent responses, while *prim2* mutants pretreated with *T. atroviride* show higher JA marker gene accumulation and lower infection by *B. cinerea*. Overaccumulation of *Ta_sRNA1* compromises ISR and increases susceptibility to necrotrophic pathogens, suggesting that *Ta_sRNA1* fine‐tunes the balance between plant growth and immunity (Fig. 1; Hernández‐Hernández *et al*.).

Phytohormones are critical regulatory nodes in the sRNA‐mediated formation of symbiotic relationships. For example, the establishment of rhizobial symbiosis in the common bean (*Phaseolus vulgaris*) is associated with *Pv*AGO5‐mediated modulation of JA‐, ethylene‐, auxin‐, and gibberellin‐dependent signalling (Sánchez‐Correa *et al*., 2022).

The ckRNAi landscape includes other sRNA classes beyond the well‐known siRNAs and miRNAs, for example tRNA fragments. Reminiscent of the situation with *T. atroviride*, similar effects of sRNAs on phytohormone balance have also been reported elsewhere: altered target gene expression is negatively associated with accumulation of tRNA fragments linked to downstream abscisic acid signalling (Huang *et al*., 2025).

In addition to computational predictions, experimental validation of ckRNAi targets such as *PRIM2* provides new insights into immune regulatory mechanisms. Root‐associated mutualists, including *T. atroviride* and mycorrhizal fungi, prime the plant immune system augmenting defence. *Ta_sRNA1*‐mediated repression of *PRIM2* also affects redox homeostasis, as shown by altered accumulation of reactive oxygen species (ROS) and pathogen‐induced cell death; this enhances resistance to *B. cinerea* by promoting systemic immune priming and sustained plant growth (Fig. 1; Hernández‐Hernández *et al*.). Histochemical staining further demonstrated increased hydrogen peroxide accumulation in *Ta_sRNA1*‐overexpressing lines, whereas *PRIM2* overexpression reduced ROS, linking *PRIM2* to suppression of ROS‐mediated defence. Similarly, *Trichoderma harzianum* mitigates *Fusarium oxysporum* infection in cucumber enhancing antioxidant enzyme activity and reducing ROS accumulation, thereby restoring redox homeostasis and potentiating host defence against Fusarium wilt (Chen *et al*., 2019). Together, these studies indicate that modulation of hormones and fine‐tuning of redox signalling by beneficial microbes through sRNA‐mediated pathways limits pathogen colonisation, reduces disease, and strengthens host resistance.

## Conclusion

The work of Hernández‐Hernández *et al*., along with emerging research on mycorrhizal and rhizobium systems, establishes ckRNAi as a fundamental mechanism in plant–microbe symbiotic relationships. RNA‐based communication is a widespread and versatile process that enables organisms to dynamically regulate their interactions. S‐genes such as *PRIM2* are silenced, plant hormones are regulated through AGO proteins, and various RNA types are used to achieve a sophisticated dialogue and to optimise symbiotic outcomes. As we continue to explain ckRNAi, we open new avenues for enhancing crop resilience and achieving sustainable agriculture by regulation of cross‐species communication.

## Disclaimer

The New Phytologist Foundation remains neutral with regard to jurisdictional claims in maps and in any institutional affiliations.
